# Anti-phospholipase A2 receptor antibodies directly induced podocyte damage *in vitro*

**DOI:** 10.1080/0886022X.2022.2039705

**Published:** 2022-03-25

**Authors:** Yanfen Li, Juntao Yu, Miao Wang, Zhao Cui, Ming-hui Zhao

**Affiliations:** a Department of Nephrology and Renal Division, Peking University First Hospital, Beijing, China; b Kintor Pharmaceutical Limited, Suzhou, China; c Peking-Tsinghua Center for Life Sciences, Beijing, China

**Keywords:** Anti-PLA2R antibody, podocyte, apoptosis, protein binding, actin

## Abstract

**Background:**

The pathogenesis of primary membranous nephropathy (MN) involves the antibodies against antigens on the cell surface of podocytes, with the majority of M-type phospholipase A2 receptor (PLA2R), and a profound podocyte dysfunction. The effects of anti-PLA2R antibodies directly to the podocytes remain unclear.

**Methods:**

Anti-PLA2R antibodies from patients with PLA2R-associated MN were affinity-purified using a column coupled with recombinant human PLA2R protein. Their effects on conditionally immortalized human podocytes were assessed by apoptosis assays, cellular calcium detection, wound healing assay, and immunofluorescent staining. Proteomics analysis was performed by LC-MS/MS and on PANTHER database.

**Results:**

The stimulation by anti-PLA2R antibodies could induce early-stage apoptosis of podocytes (MFI of Annexin *V* = 104.3 ± 19.2 vs. 36.7 ± 7.6, *p* = 0.004). The increase of calcium concentration in podocytes (MFI = 3309.3 ± 363.6 vs. 1776.3 ± 212.7, *p* = 0.015) might attribute to the endoplasmic reticulum calcium efflux. The expression of calcium/calmodulin-dependent protein kinase IV (CaMK4) was also increased (MFI = 134.4 ± 9.8 vs. 105.3 ± 10.1, *p* = 0.011). Proteomics results suggested that anti-PLA2R antibody treatment led to damage on cellular structure, and produced functional disorders on protein binding, actin filament binding, and microtubule motor activity. The staining of F-actin on foot process was reduced (MFI = 27.3 ± 2.8 vs. 47.5 ± 1.0, *p* = 0.001) and the motility and adherence capacity of podocytes were reduced (number of migrated cells = 44.7 ± 3.1 vs. 53.3 ± 4.9, *p* = 0.001) after incubation with anti-PLA2R antibodies.

**Conclusion:**

These data indicate that anti-PLA2R antibodies may directly induce podocyte damage independent of the complement system, which expands the mechanism of anti-PLA2R antibodies on MN.

## Introduction

The pathogenesis of membranous nephropathy (MN) involves *in situ* formation of subepithelial immune deposits that induce glomerular injury by damaging podocytes through complement-dependent processes [1]. Most cases of primary MN possess autoantibodies against the podocyte antigen M-type phospholipase A2 receptor (PLA2R) [2]. The antibody levels are often associated with disease activity, treatment responses, and kidney outcomes [3–5]. A recent study [6] found that anti-PLA2R IgG4 directly bound Mannan-binding lectin in a glycosylation-dependent manner, and activated the lectin pathway of the complement cascade. Assembly of the terminal C5b-9 complex and activation of the complement receptors C3aR1 or C5aR1 were required to induce proteolysis of synaptopodin and NEPH1 and podocyte injuries.

Besides the complement-induced podocyte injuries, anti-PLA2R antibodies might induce cell damage directly. PLA2R1 is widespread expressed [7], but the patients with anti-PLA2R autoantibodies do not present with any other involvements except the kidneys, suggesting that these antibodies have unique yet unidentified roles within the podocytes. Using PLA2R-transfected HEK cells and podocytes, Skoberne et al. [8] demonstrated that PLA2R enhanced cell attachment to collagen type IV and that serum with anti-PLA2R antibodies diminished podocyte adhesion. Akilesh et al. [9] found that neonatal Fc receptor (FcRn) is expressed on podocytes and functions to internalize IgG from the GBM. FcRn-deficient mice showed delayed clearance of IgG from the kidneys. Using C57Bl/6 mice immunized with the noncollagenous domain of α3 chain of type IV collagen (α3NC1), Olaru et al. [10] found that FcRn promotes the formation of subepithelial immune complexes and subsequent glomerular pathology leading to proteinuria. Ichinose et al. [11] found that the IgG derived from patients with lupus nephritis enters podocytes *via* FcRn and up-regulates the expression of calcium/calmodulin-dependent protein kinase IV (CaMK4), which is followed by increased expression of genes related to podocyte damage and T cell activation.

The effects of anti-PLA2R antibodies directly to the podocytes are unknown, neither are the possible mechanisms. In the current study, we purified anti-PLA2R antibodies from patients with PLA2R-associated MN, investigated their effects on the conditionally immortalized human podocytes and the possible molecular mechanisms. These findings may help to better understand MN pathogenesis.

## Methods

### Preparation of PLA2R protein

The extracellular portion of PLA2R1 was cloned into the HA tag-CMV-14 expression vector and transfected to HEK293 cells. After 48 h, the selection antibiotic G418 (A1720, Sigma, St Louis, USA) 800 μg/mL was added to establish a stable cell line. To generate the recombinant protein, HEK293 cells, growing in DMEM containing 800 μg/mL G418 and 10% fetal bovine serum (FBS, GIBCO, CA, USA), were collected and lyzed using FBS-free DMEM containing 50 μg/mL ascorbic acid (1043003, Sigma). The cell lysate was precipitated overnight with 3 mol/L (NH_4_)_2_SO_4_ and centrifuged for 20 min at 14,000 g. The PLA2R protein was redissolved in PBS and incubated with Pierce Anti-HA Magnetic Beads (88837, Thermo Fisher Scientific, MS, USA) for 30 min at room temperature. The bound, HA-tagged proteins were dissociated from the beads using HA peptide (26184, Thermo).

### Purification *of anti-PLA2R-specific IgG*

Circulating total IgG from plasma of MN patients with positive anti-PLA2R antibodies and healthy individuals was affinity-purified using a protein G column (GE Healthcare, Buckinghamshire, UK).

Purified PLA2R protein was coupled with CNBr-activated Sepharose 4B (GE Healthcare) to make a column for affinity purification, as described previously [12]. The column was equilibrated by 20 mmol/L Tris buffer pH 7.2 (buffer A) for five column volumes. Then the total IgG was applied to the column with buffer A for ten column volumes. Anti-PLA2R specific IgG was eluted by 0.1 mol/L glycine buffer containing 0.5 mol/L NaCl pH 2.7 for five column volumes, neutralized with 1 mol/L Tris buffer pH 8.0, and concentrated and exchanged to PBS using an Amicon® Ultra Centrifugal Filter (30 K MWCO, Merk Millipore, Temecula, CA).

#### Podocytes culturing and treatment

Conditionally immortalized human podocytes were kindly provided by Prof. Jochen Reiser (Rush University, Chicago, IL, USA). The protocol of podocytes culturing was described previously [13]. In brief, the cells were cultivated at 33 °C in the presence of RPMI-1640 medium (GIBCO), containing 10% FBS, 1% insulin-transferrin-selenium (Life Technologies, Gaithersburg, MA), and 1% Pen/Strep (Thermo). The cultured podocytes were seeded on coverslips (Thermo) and allowed to differentiate for 10 days at 37 °C in RPMI-1640 with 10% FBS and 1% Pen/Strep.

Before treatment, the medium was changed to RPMI-1640 without FBS to synchronize the cell cycle for 12 h. Anti-PLA2R specific IgG (40 μg/ml), normal human IgG (40 μg/ml, negative control), or hydroperoxide (100 μmol/L, positive control) was dissolved in FBS free RPMI-1640 and added for incubation for 24 h at 37 °C.

### *PLA2R expression and interaction with* anti-PLA2R antibodies from MN patients

The expression of PLA2R was achieved through Western-blot and immunofluorescent assay.

In Western blot assay, the podocytes were collected and incubated in RIPA buffer (P0013G, Beyotime, Nanjing, China) and protease inhibitor Cocktail (539134, Millipore, Temecula, CA) for 30 min on ice. The supernatant was collected, and SDS loading buffer was added. Podocyte whole extracts or purified PLA2R (20 μg) were electrophoresed on 12% SDS-PAGE under reducing conditions and blotted on PVDF membrane (Bio-Rad Laboratories). The membranes were blocked with 0.1% TWEEN 20 and 0.5% powdered milk in TBS 1×, then incubated by mouse anti-GAPDH (1:1000, ab8245), mouse anti-PLA2R antibodies (1:1000, ab188028), rabbit anti-PLA2R antibodies (1:1000, HPA012657, Sigma) or anti-PLA2R antibodies from MN patients. Secondary antibodies included goat anti-mouse IgG H&L (HRP) (ab205719, Abcam), goat anti-rabbit IgG H&L (HRP) (ab205718, Abcam) and goat anti-human IgG H&L (HRP) (ab6858, Abcam).

In the immunofluorescent assay, the podocytes were fixed with 4% paraformaldehyde for 15 min. After washing thrice, the cells were incubated with mouse anti-PLA2R antibodies (ab188028, Abcam) at 1:100 for 1 h at 37 °C and then CY3 conjugated goat anti-mouse antibody at 1:250 for 1 h at 37 °C. The cells were washed thrice for fluorescent microscopy (40x and 63x, Leica DFC7000 T) observation.

All negative controls were performed by omitting primary antibodies.

#### Apoptosis detection assay

The commercial FITC Annexin V Apoptosis Detection Kit with 7-AAD (640922, Biolegend) was used to evaluate the apoptosis of podocytes. After the above treatments, the podocytes were washed twice and resuspended in Annexin V binding buffer at a concentration of 0.25–1.0 × 10^7^ cells/ml. The cell suspension 100 µl was added with FITC Annexin V 5 µl and 7-AAD Viability Staining Solution 5 µl. The cells were incubated for 15 min at room temperature in the darkness and then analyzed by flow cytometry (BD FACS Verse, New Jersey, USA) and FlowJo software (v10.8). The cells in the lower right quadrant were classified as exhibiting early-stage apoptosis (labeled by FITC-conjugated Annexin V), and the cells in the upper right quadrant were identified as exhibiting late-stage apoptosis (labeled by 7-AAD).

#### Detection of calcium concentration

Fluorescent calcium indicator fluo-4 (F14201, Thermo) was used to define the level of cellular calcium through flow cytometry and fluorescent microscopy.

The podocytes were collected and washed twice with PBS or FBS-free RPMI-1640 after the above treatments. Fluo-4 DMSO stock was dissolved in PBS to a final concentration of 1 μmol/L. The cells were resuspended by PBS with fluo-4 and incubated for 30 min at 37 °C in the darkness. After incubation, the cells were washed twice by fluo-4 free PBS and analyzed by flow cytometry or fluorescent microscopy (20 x, DFC7000 T, Leica).

#### Proteomic studies of podocytes

The podocytes were incubated in ammonium bicarbonate (A6141, Sigma) with 6 mol/L urea and 25 μmol/L DTT (10197777001, Sigma) for cell disruption and protein reduction. Iodoacetamide (Sigma) was added at 100 μmol/L as alkylating sulfhydryl reagent. The peptides were prepared by Trypsin (V528A, Promega, Madison, USA) in the ratio of 1:50 (protein: Trypsin) at 37 °C for 16 h.

The samples were evaluated by Q-Exactive hybrid quadrupole-Orbitrap mass spectrometer (Thermo). Peptide and protein information was retrieved by Mascot and Proteome Discoverer 2.0 (Thermo), based on UniProt-human.Fasta database (www.uniprot.org). The gene ontology (GO) analysis was realized by R 3.4.4 (cran.r-project.org) based on Protein ANalysis THrough Evolutionary Relationships (PANTHER) database (www.pantherdb.org). Fisher’s exact test, with the Benjamini–Hochberg false discovery rate (FDR) correction is used for the over-representation test.

#### Wound healing assay

One cross scratch was performed with a 200 μL sterile tip in each well of the plates. The podocytes were treated as above and the scratches were observed on the microscope (20×, ECLIPSE TS 100, Nikon) at 0 h and 16 h. The number of cells that migrated into the same-sized scratch with the majority of cell body were counted for each well.

#### Staining of F-actin by immunofluorescence

The podocytes were fixed by 4% paraformaldehyde, permeabilized by 0.1% TritonX-100 (T8787, Sigma) for 10 min on ice, and incubated with rhodamine-phalloidin (ab235138, Abcam) (1:100) for 1 h at room temperature. The cells were observed on a confocal microscope (20 x, LSM 780, Zeiss). ImageJ is used for measuring the mean fluorescent intensity (MFI) of whole field cells (20 × objective) from three independent assays.

#### CaMK4 expression

After treatment of normal human IgG and anti-PLA2R IgG, the podocytes were collected for Western blot assay as described ahead. Primary and secondary antibodies were incubated with mouse anti-human CaMK4 (sc-166156, Santa Cruz) and goat anti-mouse IgG H&L (HRP) (ab205719, Abcam).

#### Statistics

All the experiments were performed at least three times. For data of normal distribution, the results were expressed as mean ± standard deviation (SD). For data of unnormal distribution, the results were expressed as median (interquartile range, IQR). The differences were assessed using one-way analysis of variance (ANOVA) followed by Tukey’s *post hoc* analysis or Mann-Whitney U test as indicated. The data were analyzed using SPSS software, version 24.0 (IBM, New York, USA). A P value < 0.05 was considered significant.

## Results

### PLA2R expression on podocytes and its binding with anti-PLA2R antibodies

**Figure 1.**
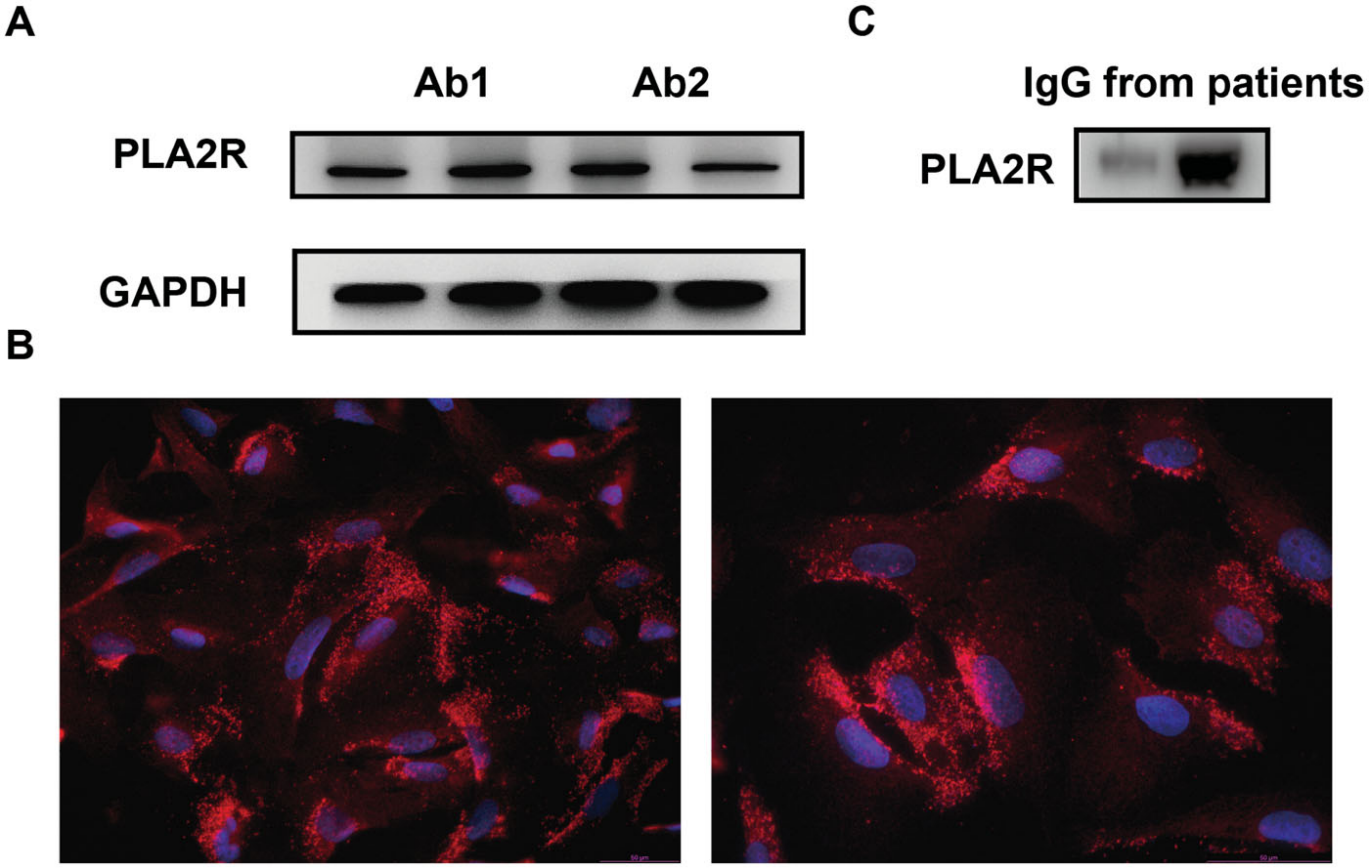
PLA2R expression on cultured human podocytes and its binding with anti-PLA2R antibodies from MN patients. Western blot (A) and immunofluorescence (B) assay showed the expression of PLA2R on conditionally immortalized human podocytes. Ab1: mouse monoclonal anti-PLA2R antibodies; Ab2: rabbit polyclonal anti-PLA2R antibodies. (C) Anti-PLA2R antibodies from MN patients blotted with PLA2R from both cultured podocytes (left band) and purified PLA2R protein (right band).

Before conducting podocyte assays, we assessed the expression of PLA2R on cultured human podocytes. PLA2R expression was shown by Western blot assay and immunofluorescence staining (Figures 1(A,B)). Western blot revealed the blots of two commercial anti-PLA2R antibodies on cultured human podocytes. Immunofluorescence staining showed that PLA2R was expressed on the podocyte cell surface. The binding of anti-PLA2R antibodies from MN patients with PLA2R from cultured human podocytes was shown by Western blot (Figure 1(C)).

### Anti-PLA2R antibodies could induce podocyte apoptosis

To examine apoptotic podocytes, the early-stage (Annexin V+) and late-stage (7-AAD+) of apoptosis were detected after the treatments by anti-PLA2R antibodies.

**Figure 2.**
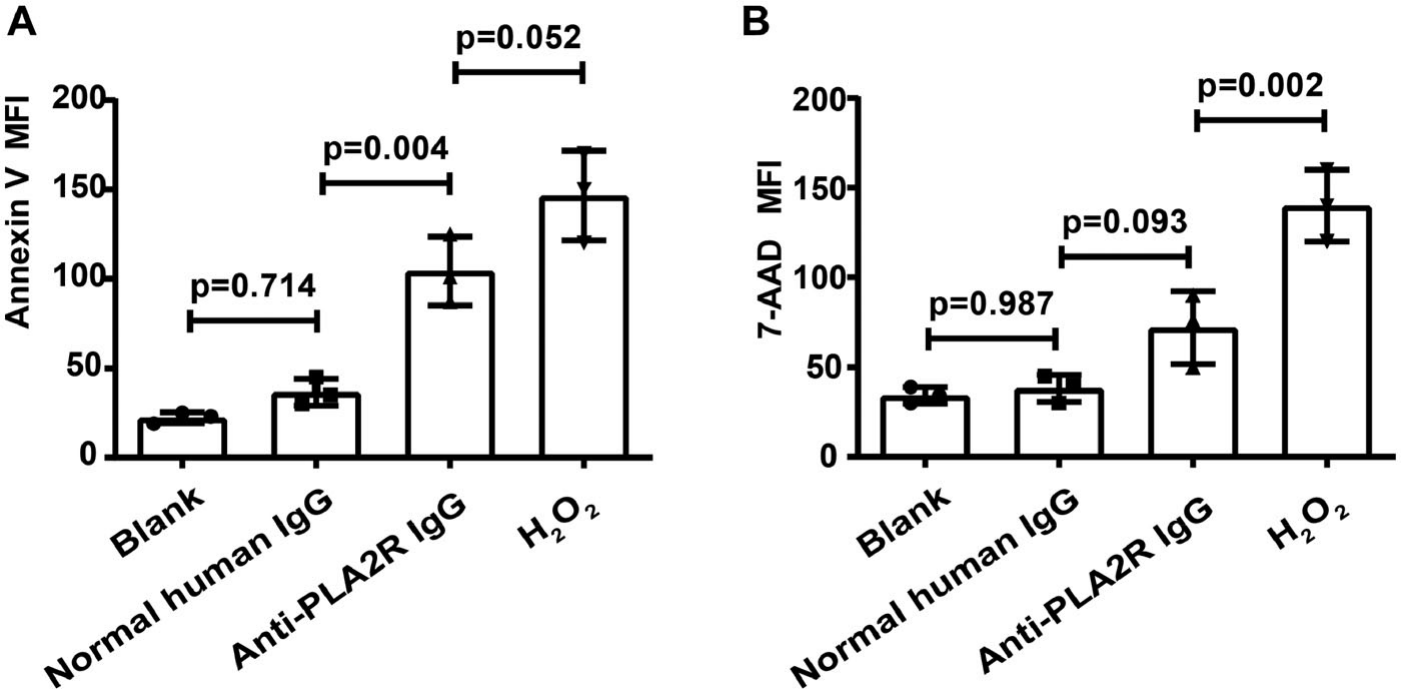
Anti-PLA2R antibodies induced apoptosis of human podocytes. (A) After treatment with anti-PLA2R antibodies for 24 h, the concentration of Annexin V (early stage of apoptosis) of human podocytes was increased significantly. (B) The concentration of 7-AAD (late stage of apoptosis) was increased as well but not reaching significance.

The level of Annexin V + was significantly increased after treatment with anti-PLA2R antibodies at 40 μg/ml for 24 h, compared to the group treated with normal human IgG (MFI = 104.3 ± 19.2 vs. 36.7 ± 7.6, *p* = 0.004) (Figure 2(A)), indicating the beginning of cell apoptosis.

The level of cellular-binding 7-AAD in the anti-PLA2R antibody treatment group was increased compared to the group treated with normal human IgG but not reaching significance (MFI = 72.0 ± 20.3 vs. 38.3 ± 7.6, *p* = 0.093) (Figure 2(B)), which indicated the late stage of apoptosis and process of cell death.

### Anti-PLA2R antibodies increased the intracellular calcium of podocytes

**Figure 3.**
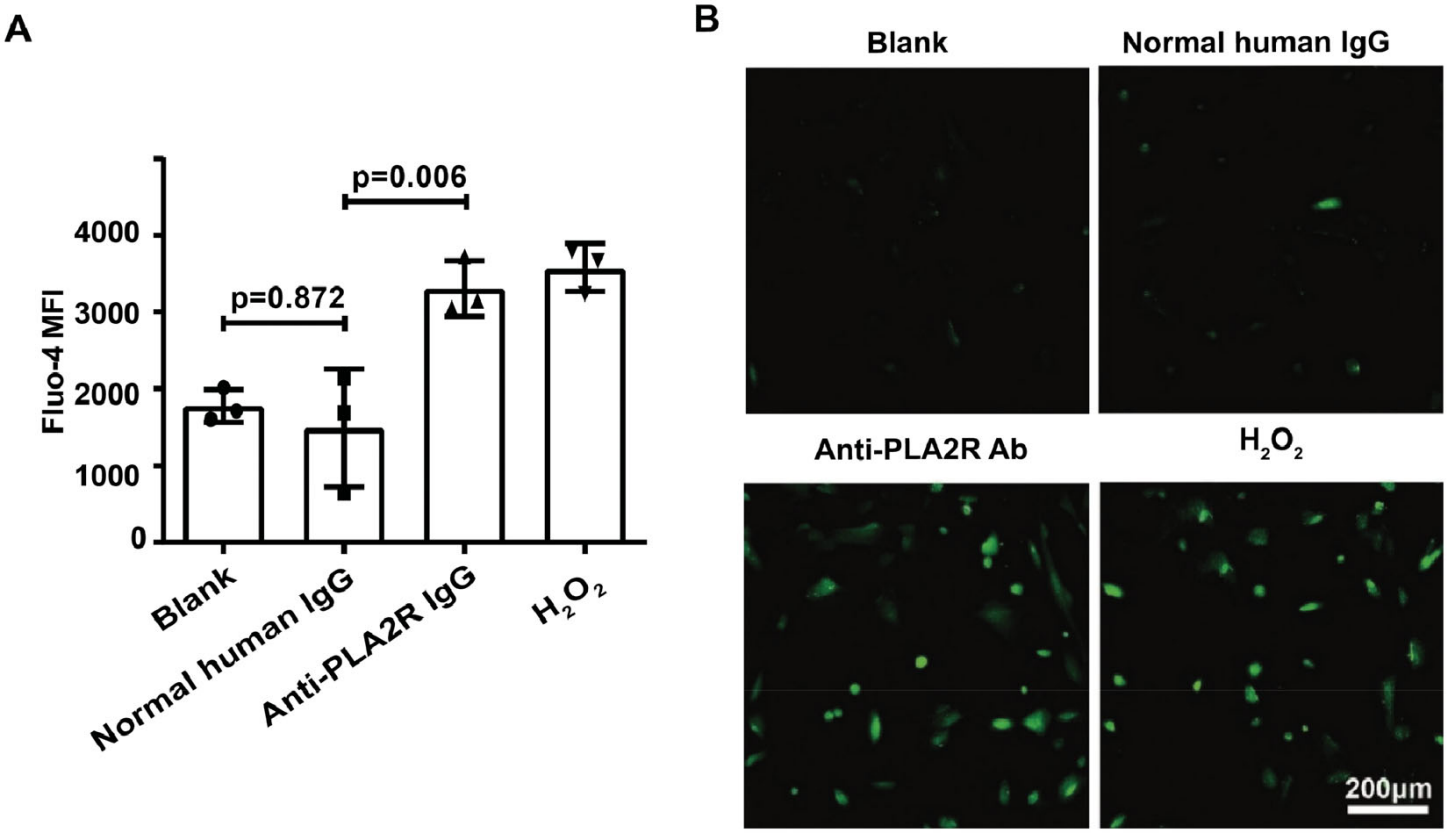
Anti-PLA2R antibodies increased the intracellular calcium of human podocytes. The stimulation of anti-PLA2R antibodies increased the concentration of cellular calcium in podocytes, through the observation of FACS (A) and immunofluorescence (B).

After treatment of anti-PLA2R antibodies, the levels of intracellular calcium were significantly ascendant (MFI = 3309.3 ± 363.6 vs.1776.3 ± 212.7, *p* = 0.015) in the podocytes, while the treatment of human normal IgG could not cause the increment of cellular calcium (MFI = 1492.3 ± 764.5 vs. 1776.3 ± 212.7, *p* = 0.872) (Figure 3). Since no overdosed calcium participated in this system, the increasing level of cellular calcium indicated endoplasmic reticulum calcium released. This result illustrated that anti-PLA2R antibodies could trigger endoplasmic reticulum calcium efflux.

### Proteomics findings after anti-PLA2R antibody treatment

**Figure 4.**
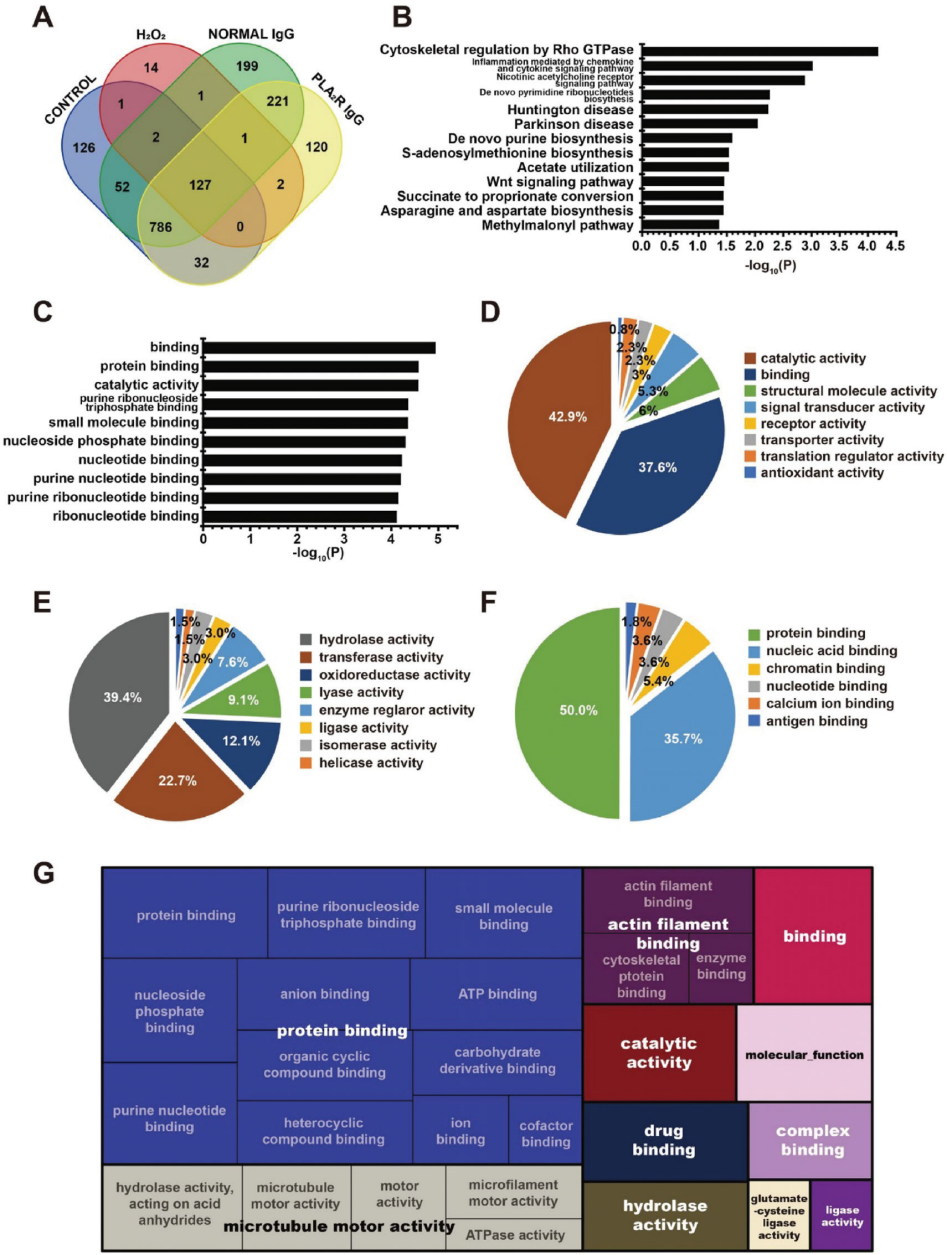
Proteomics study of podocytes. (A) Venn diagram of logical relations among proteins of podocyte after treatment with PBS, H_2_O_2_, human normal IgG, and anti-PLA2R antibodies. There were in total 1684 unique proteins detected. The protein number of each set was 1126 in the PBS treated group (blank control), 148 in the H_2_O_2_ treated group (positive control), 1389 in the normal human IgG treated group (negative control), and 1289 in the anti-PLA2R antibody treated group. (B) The pathway annotation of the 120 specific proteins (*p*< 0.05) in the anti-PLA2R antibody treated group was shown in the column. (C) The GO annotation in molecular function of the 120 specific proteins, with the top 10 significantly enriched terms shown in column. (D) The pie chart of GO annotation cluster in molecular function of the 120 specific proteins in anti-PLA2R antibody treated group. (E) The pie chart of GO annotation cluster in molecular function of the 57 specific proteins hit catalytic activity category in anti-PLA2R antibody treated group. (F) The pie chart of GO annotation cluster in molecular function of the 50 specific proteins hit binding category in anti-PLA2R antibody treated group. # In D–F, the percentage showed the percent of gene hit against Function Database hits. (G) The treemap of GO annotation in molecular function of the 120 specific proteins in anti-PLA2R antibody treated group. The nested rectangles displayed the hierarchical relationship of different terms. The scales of rectangles were defined by the − log_10_ (*p*-value) of each category.

To identify the possible molecular mechanisms, anti-PLA2R antibody treated podocytes and control cells were lysed, separated by SDS-PAGE, and analyzed by LC-MS/MS after in-gel tryptic digestion. There were in total 1684 unique proteins detected, with 1126 in the non-treated group, 148 in hydroperoxide treated group, 1389 in the human normal IgG treated group, and 1289 in the anti-PLA2R antibody treated group. There were 126 proteins specific in the non-treated group, 14 proteins specific in the hydroperoxide treated group, 199 proteins specific in the human normal IgG treated group, and 120 proteins specifics in the anti-PLA2R antibody treated group (Figure 4(A)).

Further analysis was processed on the 120 specific proteins in the anti-PLA2R antibody treated group. After Protein ANalysis THrough Evolutionary Relationships (PANTHER) pathway annotation enrichment analysis, 13 pathway terms presented significant fold enrichment (*p*< 0.05). All these categories were overloaded fold enrichment ratio (FER) >1.5. The category with the lowest p-value was the cytoskeletal regulation by Rho GTPase pathway (*p* = 6.87 × 10^−5^, FER = 9.22) (Figure 4(B)).

Functional classification on gene ontology molecular function (GO-MF) of these 120 specific proteins was presented in Figures 3(C–G). There were 163 genes that participated in the expression of 120 specific proteins. 133/163 genes hit the GO-MF database and were involved in 4660 categories in GO-MF, while 37/4660 categories presented significant fold enrichment (*p*< 0.05). The top 10 categories with the lowest p-value were shown in Figure 3(C). There were 24/37 categories related to binding. Considering the number of gene hits, the categories of catalytic activity and binding constituted more than 80% of GO-MF functional database hits (catalytic activity: 57/133, 42.9%; binding: 50/133, 37.6%) (Figure 4(D)). The categories of hydrolase activity (26/66, 39.4%) and transferase activity (15/66, 22.7%) constituted more than 60% of the functional hits in catalytic activity (Figure 4(E)). The categories of protein binding (28/56, 50.0%) and nucleic acid binding (20/56, 35.7%) constituted more than 80% of the functional hits in binding (Figure 4(F)). The hierarchical relationship of all 133 GO-MF-relevant genes was illustrated in the treemap (Figure 4(G)). The scales of rectangles were defined by the -log_10_ (*p*-value) of each entry. The root entries of protein binding, actin filament binding, and microtubule motor activity were the major components in GO-MF categories, which suggested that there might be disorder in the function of cytoskeleton structure, cell junction, migration, and signal transduction, after the treatment of anti-PLA2R antibodies on podocytes.

#### Anti-PLA2R antibodies impeded the cellular motility and adhesion of podocytes

**Figure 5.**
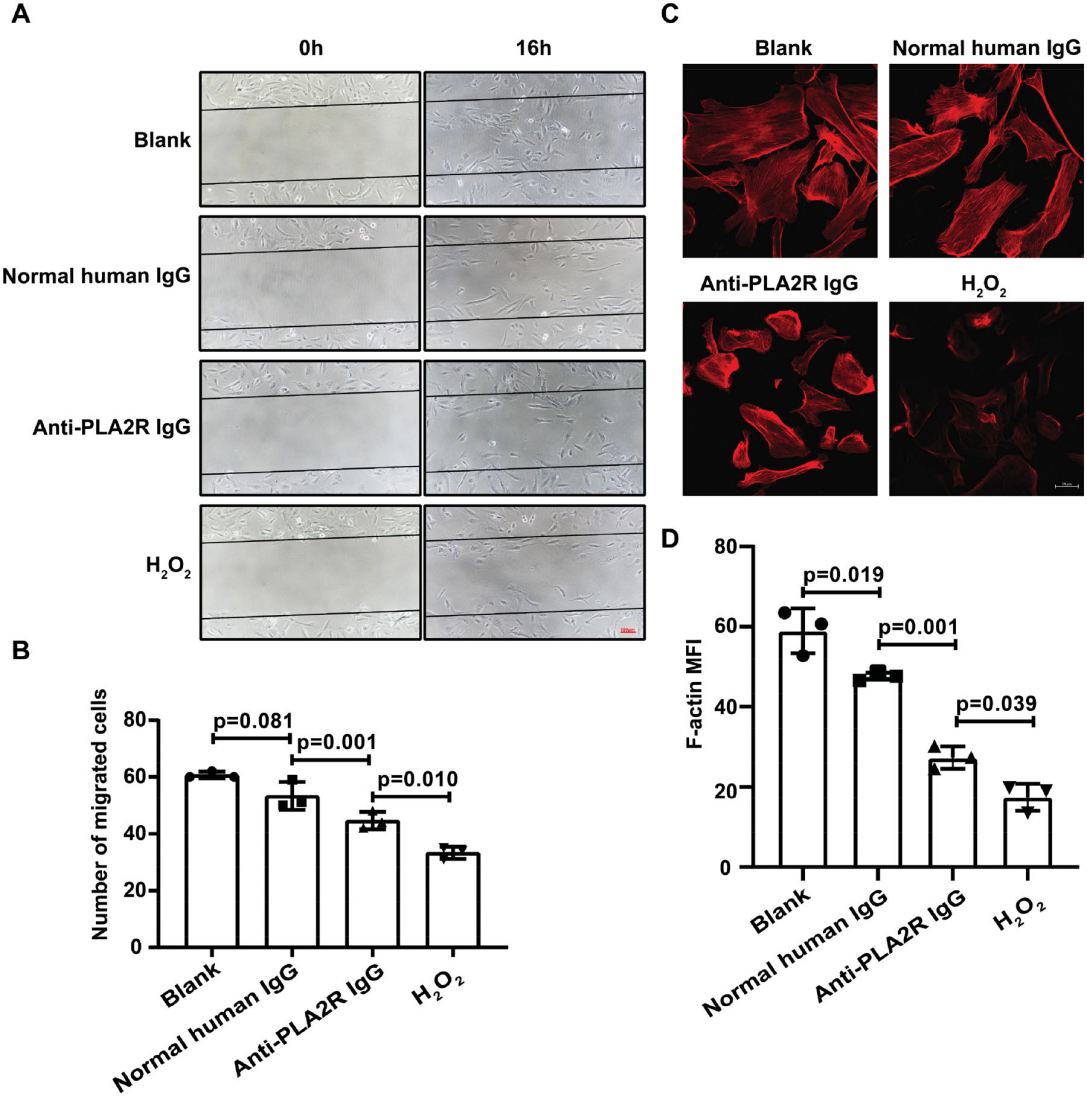
Anti-PLA2R antibodies impeded the cellular motility and adhesion of podocytes. In wound healing assay, the number of cells that migrated into the same-sized scratch was significantly reduced after the treatment of anti-PLA2R antibodies for 16 h (A, B). The staining of F-actin stress fibers was reduced as well (C, D).

Podocyte motility was examined by a wound-healing assay after the treatment of anti-PLA2R antibodies. The number of podocytes that migrated into the same-sized scratch was significantly decreased (number of migrated cells = 44.7 ± 3.1 vs. 53.3 ± 4.9, *p* = 0.001), compared to that in the normal human IgG treated group (Figures 5(A,B)).

Actin reorganization is regarded as a symbol of podocyte foot process effacement in the experiments *in vitro*. After the treatment of anti-PLA2R antibodies, the staining of F-actin stress fibers was significantly reduced on the podocytes (MFI = 27.3 ± 2.8 vs. 47.5 ± 1.0, *p* = 0.001) (Figures 5(C,D)).

#### Anti-PLA2R antibodies increased the protein expression of CaMK4 in vitro podocytes

**Figure 6.**
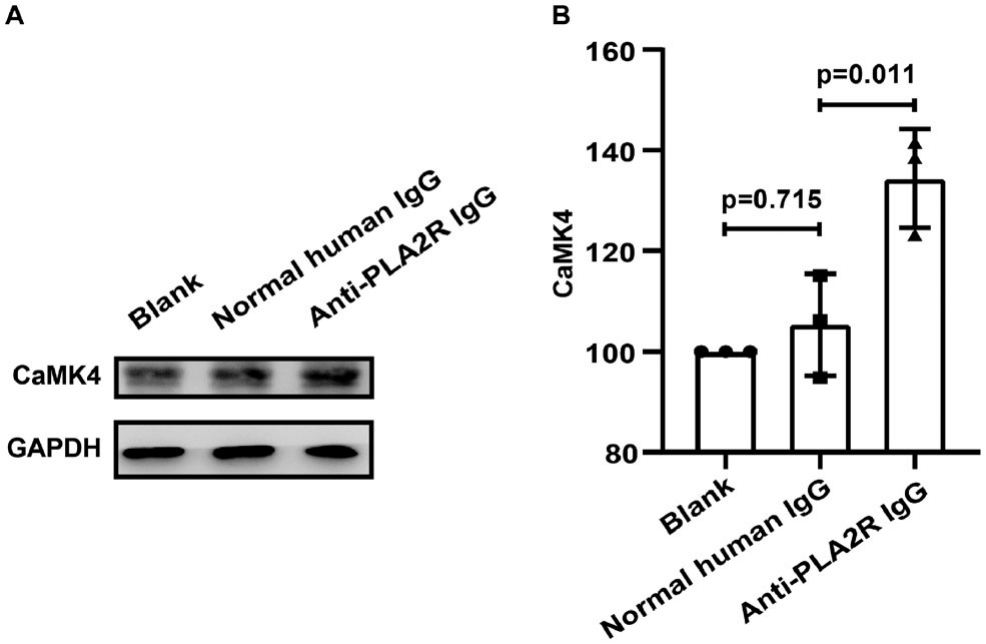
Anti-PLA2R antibodies increased the protein expression of CaMK4 of podocytes. After the treatment of anti-PLA2R antibodies, CaMK4 was significantly increased on podocytes, compared to both blank control and normal human IgG control, as shown in Western blot.

CaMK4 is activated after phosphorylation on a threonine residue by the upstream of Ca2+/CaM-dependent kinase kinases [14,15]. After the treatment of anti-PLA2R antibodies, the protein expression of CaMK4 on the podocytes was significantly increased (MFI = 134.4 ± 9.8 vs. 105.3 ± 10.1, *p* = 0.011) (Figures 6(A,B)).

## Discussion

In the current study, we demonstrated that MN patients derived anti-PLA2R antibodies could trigger early-stage apoptosis on podocytes, induce endoplasmic reticulum calcium efflux, lead to disorders of protein binding system in podocytes, and result in the loss of F-actin on foot process and the reduced capacity of cell motility. These findings indicated that anti-PLA2R antibodies may induce podocyte damage directly, independent of the complement activation, which provides more information for the in-depth understanding of MN pathogenesis.

Our data firstly presented that anti-PLA2R antibodies translocated the phosphatidylserine from the intracellular leaflet to the external leaflet, which acts as an activator of protein kinase C (PKC). Atypical PKC (aPKC) associates with the Neph1-nephrin complex and localizes to the leading edge and filopodia tips of cultured podocytes. Huber et al. [16] have demonstrated that podocyte-specific deletion of aPKCλ/ι in mice results in slit diaphragm displacement, foot process effacement, proteinuria, and accelerated renal failure. aPKC-deficient podocytes failed to form the normal network of foot processes, leading to defective glomerular maturation with incomplete capillary formation and mesangiolysis [17]. The reduced phosphatidylserine inside podocytes by anti-PLA2R antibodies might result in a defective aPKC signaling, which leads to the foot process effacement and proteinuria.

We found that anti-PLA2R antibodies mediated endoplasmic reticulum calcium efflux in podocytes, which might induce podocyte damage. Ichinose [11] previously reported that lupus nephritis derived IgG enters podocytes *via* the FcRn and up-regulates Ca2+/calmodulin-dependent kinase (CaMK) and downstream genes involved in podocyte injury. Targeted delivery of a CaMK4 inhibitor to podocytes preserved their ultrastructure, averted immune complex deposition and crescent formation, and suppressed proteinuria in lupus-prone mice, despite systemic autoimmunity remaining intact. They further demonstrated that CaMK4 expression is increased in podocytes of patients of active lupus nephritis and transplant glomerulopathy. We found that anti-PLA2R IgG enhanced the expression of CaMK4 on human podocytes, which is consistent with the previous findings. The primary source of renal reactive oxygen species, particularly H_2_O_2_, is NADPH oxidase 4 (NOX4). Ilatovskaya et al. [18] found that NOX4-derived H_2_O_2_ contributes to podocyte damage in diabetic kidney disease *via* elevation of podocyte calcium. The diabetic rats^Nox4-/-^ exhibited significantly lower basal intracellular Ca2+ levels in podocytes and less damage. The angiotensin II-elicited calcium flux was blunted in glomeruli isolated from diabetic rats^Nox4-/-^. H_2_O_2_ stimulated TRPC-dependent calcium influx in podocytes, which was blunted in podocytes from *Trpc6*-knockout mice. Several studies [19–21] demonstrated that the increment of cytoplasmic calcium concentration resulted in calcium uptake by mitochondria accompanied by the simultaneous release of cytochrome c, which then induced apoptosome formation and caspases activation.

To further explore the possible molecular mechanism of anti-PLA2R antibodies on podocytes, we compared the proteomics data of anti-PLA2R antibody treated podocytes and that of control cells. The results showed that the treatment of anti-PLA2R antibodies produced up-regulation on protein binding, actin filament binding, and microtubule motor activity. The accumulation of malfunctioning structural proteins evokes endoplasmic reticulum stress in podocytes [22], which might induce the calcium efflux and early-stage apoptosis found in the current study. Cytoskeleton regulation of Rho GTPase was the most distinctive pathway. Actin is the key component of podocyte foot processes in comparison with the microtubule-based primary and secondary processes [23]. The changes of actin cytoskeleton initiate an active procedure of foot process effacement, which causes the loss of cell adherence capability [24,25].

In line with these thoughts, we investigated the adherence and motility of podocytes using the wound healing assay and stained the F-actin of podocytes. We found that the migration of podocytes was impeded accompanied by the reduction of stress fibers, after the treatment of anti-PLA2R antibodies. These findings give evidence to the morphologic and motility changes of podocytes and were consistent with the findings of proteomics data. Podocyte loss is an important component of disease progression in MN [26]. The potential causes of podocyte loss are podocyte detachment and apoptosis [27]. Several factors could implement podocyte detachment, such as changes in the actin cytoskeleton, foot process effacement, and alteration of slit diaphragm integrity [28]. According to our study, both podocyte F-actin reconstruction and apoptosis might participate in the progress of PLA2R-related MN.

The high expression of PLA2R1 is also found to increase podocyte apoptosis in the kidneys of MN patients [29]. PLA2R1 ectopic overexpression in originally PLA2R1 negative cancer cells induces apoptosis and onco-suppressing role independently of sPLA2 binding [7,30–33]. However, we did not find any change in PLA2R expression on podocytes after its antibody’s stimulation.

In conclusion, these findings provide a feasible avenue to explain the potential pathogenicity of anti-PLA2R antibodies on MN, by directly inducing the construction alteration and apoptosis of podocytes. Further investigations are required to elucidate signaling pathways of antibody-mediated disorders.

## Supplementary Material

Supplemental Material
